# Special Issue: Protein–Protein Interactions: New Perspectives in Drug Discovery

**DOI:** 10.3390/ijms262311671

**Published:** 2025-12-02

**Authors:** Tibor Szénási

**Affiliations:** HUN-REN Research Center for Natural Sciences Budapest, 1117 Budapest, Hungary; szenasi.tibor@ttk.hu

Protein–protein interactions (PPIs) are the cornerstone of cellular life, forming a vast and dynamic network—the “interactome”—that governs nearly every biological process, from signal transduction and DNA replication to metabolic regulation and immune responses [1]. Given their central role, it is unsurprising that the dysregulation of these interactions is a hallmark of numerous human pathologies, including cancer, neurodegenerative disorders, infectious diseases and pulmonary fibrosis [2]. Consequently, the interfaces of PPIs have emerged as a highly attractive, albeit challenging, class of therapeutic targets [3].

For decades, the field of drug discovery was dominated by the pursuit of inhibitors for enzymes and receptors, which possess well-defined “grooved” active sites. In contrast, PPIs were often deemed “undruggable” due to their large, flat, and often transient interaction surfaces, which present significant challenges for high-affinity binding by traditional small molecules [4].

However, the last two decades have witnessed a paradigm shift. This shift has been driven by a deeper understanding of PPI structures, the identification of “hotspots” that dominate binding energy, and, crucially, the development of sophisticated methodologies to study and modulate these complex interactions [5].

The methodological toolkit for investigating PPIs has expanded significantly. Classical in vivo and in vitro techniques, such as the yeast two-hybrid (Y2H) system [5] and pull-down assays [6], remain invaluable for initial screening. These have been complemented by powerful cell-based assays that provide critical context in a living system, including Bimolecular Fluorescence Complementation (BiFC) [7,8] and ELISA-based methods [8,9], as well as Bioluminescence Resonance Energy Transfer (BRET) [10]. More recently, next-generation split-luciferase systems, such as NanoBiT (nanosplit), have emerged, offering unprecedented sensitivity in real-time complementation assays [11,12].

Furthermore, the advent of computational and digital methods has revolutionized the field. Structure-based drug design, enhanced by artificial intelligence (AI) and the predictive power of tools like AlphaFold-Multimer, now allows for the in silico screening and rational design of PPI inhibitors with greater speed and accuracy [13,14]. This integration of computational biology with biophysical validation is unlocking previously inaccessible targets and paving the way for novel therapeutic modalities, such as molecular glues [15].

This Special Issue, “Protein–Protein Interactions: New Perspectives in Drug Discovery”, aimed to capture the momentum in this exciting field, highlighting recent breakthroughs in methodology, target identification, and the development of novel inhibitors.

I am pleased to present a collection of three outstanding contributions that span the breadth of modern PPI research. The contributions begin with a critical analysis of a pressing global health challenge. Alejandra Garcia-Aguilar et al. provided an “In Vitro Analysis of SARS-CoV-2 Spike Protein and Ivermectin Interaction”. Given that the Spike (S) protein of SARS-CoV-2 is a key molecular target for developing drug therapies against COVID-19, their work investigates the interaction between the S protein and the host cell receptor, contributing to the urgent search for novel treatments.

Broadening the scope to disease regulation, Meishen Ren et al. contributed a comprehensive review titled “Connective Tissue Growth Factor: Regulation, Diseases, and Drug Discovery”. Their work details how selecting targeted molecules, such as the Connective Tissue Growth Factor (CTGF/CCN2), is crucial for drug efficacy. The review provides an essential overview of this target’s role in the progression of numerous diseases.

Finally, focusing on the modulation of complex, PPI-driven signaling pathways, Zhou et al. identify a novel 6-methoxybenzofuran compound that promotes osteoblast differentiation. Their work carefully elucidates the role of the BMP2–ERK–ATF4 axis, providing a valuable example of how modulating a signaling cascade—which is itself governed by sequential protein interactions—can lead to potent therapeutic effects, in this case for treating bone loss.

Together, these three articles underscore the remarkable progress and dynamism of the PPI field. The “undruggable” dogma is being dismantled, replaced by a sophisticated, multi-disciplinary approach that integrates AI-driven prediction, innovative HTS methodologies, and the rational design of diverse modalities. The path from interactome mapping to clinical application is becoming clearer, promising a new generation of therapeutics for diseases that have, until now, eluded effective treatment.

I extend my sincere gratitude to all the authors who contributed their valuable research and insights to this Special Issue. I am also deeply grateful to the peer reviewers for their diligent efforts and constructive feedback, which were essential in ensuring the high quality of the published articles. I hope that this collection will serve as a valuable resource and inspire further innovation in the ongoing quest to drug the interactome.
